# Clotting Beyond Boundaries: Extensive Iliocaval Thrombosis Revealing Homozygous Factor V Leiden Mutation in a Young Adult Following Prolonged Air Travel

**DOI:** 10.7759/cureus.116476

**Published:** 2026-09-18

**Authors:** Dikshya Yadav, Edip Katayifci, Falak Naz, David Remyes, Jay Nfonoyim

**Affiliations:** 1 Internal Medicine, Richmond University Medical Center, Staten Island, USA; 2 Radiology, Richmond University Medical Center, Staten Island, USA; 3 Research, Richmond University Medical Center, Staten Island, USA; 4 Medicine, New York Institute of Technology (NYIT) College of Osteopathic Medicine, Staten Island, USA; 5 Pulmonary and Critical Care, Richmond University Medical Center, Staten Island, USA

**Keywords:** air travel-associated thrombosis, deep vein thrombosis, factor v leiden mutation, iliocaval thrombosis, inherited thrombophilia, venous thromboembolism

## Abstract

Extensive iliocaval thrombosis is a rare manifestation of venous thromboembolism and should prompt evaluation for inherited thrombophilia in young patients. We report a previously healthy 21-year-old man with a homozygous factor V Leiden mutation who presented with bilateral groin pain, fever, and syncope two weeks after a 12-hour international flight. Imaging demonstrated extensive thrombosis involving the infrarenal and intrahepatic inferior vena cava, the bilateral common and external iliac veins, and the right renal vein with extensive collateralization. He underwent staged mechanical thrombectomy and balloon venoplasty, followed by indefinite anticoagulation. This case highlights the importance of recognizing atypical presentations of extensive iliocaval thrombosis, the synergistic risk of homozygous factor V Leiden mutation and prolonged immobility, and the need for individualized multidisciplinary management.

## Introduction

Thrombosis is a major cause of morbidity and mortality worldwide and is responsible for conditions including myocardial infarction, stroke, and venous thromboembolism (VTE), which encompasses deep vein thrombosis (DVT) and pulmonary embolism [1]. Most DVTs involve the deep veins of the lower extremities; however, thrombus may extend proximally into the iliac veins and inferior vena cava (IVC), resulting in iliocaval thrombosis. IVC thrombosis is uncommon, accounting for fewer than 5% of DVT cases, and may be underrecognized because of its variable and nonspecific clinical presentation [2,3]. The IVC is the major venous conduit returning blood from the lower body to the right atrium, while the iliac veins drain the lower extremities and pelvis into the IVC. Thrombosis involving these vessels can result in extensive venous obstruction and impaired venous return, with complications including pulmonary embolism, recurrent thrombosis, chronic venous insufficiency, post-thrombotic syndrome, and long-term disability [2-4].

The development of iliocaval thrombosis is multifactorial and reflects the interaction of venous stasis, endothelial injury, and hypercoagulability. Important risk factors include prolonged immobilization, malignancy, inflammatory conditions, inherited thrombophilia, and congenital abnormalities of the IVC or venous anatomy [2-4]. Among inherited thrombophilias, factor V Leiden (FVL) is the most common inherited thrombophilia associated with venous thrombosis among individuals of European ancestry. FVL results from a mutation in the factor V gene that causes resistance to inactivation by activated protein C, thereby prolonging factor Va activity and increasing thrombin generation [5,6]. Although heterozygous FVL is relatively common, homozygous FVL is uncommon and is associated with a substantially higher risk of VTE than heterozygosity. Individuals with homozygous FVL may develop thrombosis at a younger age and may be more likely to experience extensive or recurrent venous thrombosis [5].

Prolonged air travel is an established transient risk factor for VTE, with prolonged sitting and limited mobility contributing to venous stasis [7,8]. This risk may be further increased in individuals with an underlying thrombophilic disorder. Current guidelines emphasize that thrombophilia testing should be considered selectively when the results are likely to influence clinical management or counseling, rather than performed routinely in all patients with VTE [9,10]. In patients with VTE at a young age, thrombosis at an unusual site, or a strong personal or family history of thrombosis, identification of an inherited thrombophilia may provide clinically relevant information for recurrence risk assessment, family counseling, and decisions regarding anticoagulation [9,10].

We report the case of a previously healthy 21-year-old man with homozygous FVL who developed extensive iliocaval thrombosis involving the intrahepatic IVC, bilateral iliac veins, and right renal vein following prolonged international air travel. Extensive collateral venous circulation and limited thrombus retrieval during endovascular intervention suggested a possible acute-on-chronic thrombotic process. This case highlights the importance of considering inherited thrombophilia in young patients presenting with extensive or atypical venous thrombosis and illustrates the diagnostic and therapeutic challenges associated with extensive iliocaval thrombosis.

## Case presentation

A 21-year-old previously healthy man presented with progressively worsening bilateral groin pain, intermittent fever, and a recent syncopal episode. His family history was significant for FVL mutation, with both parents confirmed to be heterozygous carriers. He had no prior history of VTE, had never undergone thrombophilia testing, and had no history of surgery, trauma, malignancy, or other chronic medical conditions. His siblings had no known history of VTE or thrombophilia.

Two weeks before presentation, the patient returned to the United States from Egypt on a nonstop 12-hour commercial flight. Four days after travel, he developed mild right-sided groin discomfort that progressively worsened over several days. He initially attributed his symptoms to a musculoskeletal injury and remained ambulatory. Eight days after travel, he experienced a transient syncopal episode while standing at home without preceding chest pain, palpitations, or seizure-like activity. He was evaluated at an outside emergency department, where contrast-enhanced computed tomography angiography (CTA) of the abdomen and pelvis was initially interpreted as unremarkable. Routine laboratory studies and urinalysis were reportedly unrevealing. His symptoms were attributed to a nonspecific illness, and he was discharged with conservative management. On retrospective review of the initial CTA, however, extensive thrombosis involving the IVC, bilateral iliac veins, and pelvic venous system was identified, demonstrating that the thrombotic process was already present at the initial evaluation.

Over the following week, the patient developed persistent intermittent fevers, progressive bilateral groin pain, and increasing difficulty with ambulation, prompting presentation to our institution for further evaluation.

On arrival, he appeared uncomfortable but was alert and oriented. His temperature was 101.5°F (38.6°C), heart rate 110 beats/min, blood pressure 102/52 mmHg, respiratory rate 17 breaths/min, and oxygen saturation 98% on room air. Physical examination revealed bilateral lower extremity discomfort with minimal edema. Peripheral pulses were intact and symmetric, with no significant erythema, skin discoloration, palpable cords, or evidence of limb ischemia. Cardiopulmonary examination was otherwise unremarkable.

An extensive infectious evaluation, including blood cultures, urine cultures, cerebrospinal fluid analysis, and viral testing, was negative. Baseline coagulation studies showed a mildly prolonged prothrombin time of 15.7 seconds and an international normalized ratio (INR) of 1.30, with a normal activated partial thromboplastin time of 31.7 seconds. The D-dimer level was markedly elevated at 5.46. Fibrinogen was not measured. Given the persistent fever and progressive groin pain without an identifiable infectious source, further vascular evaluation was pursued.

**Figure 1:**
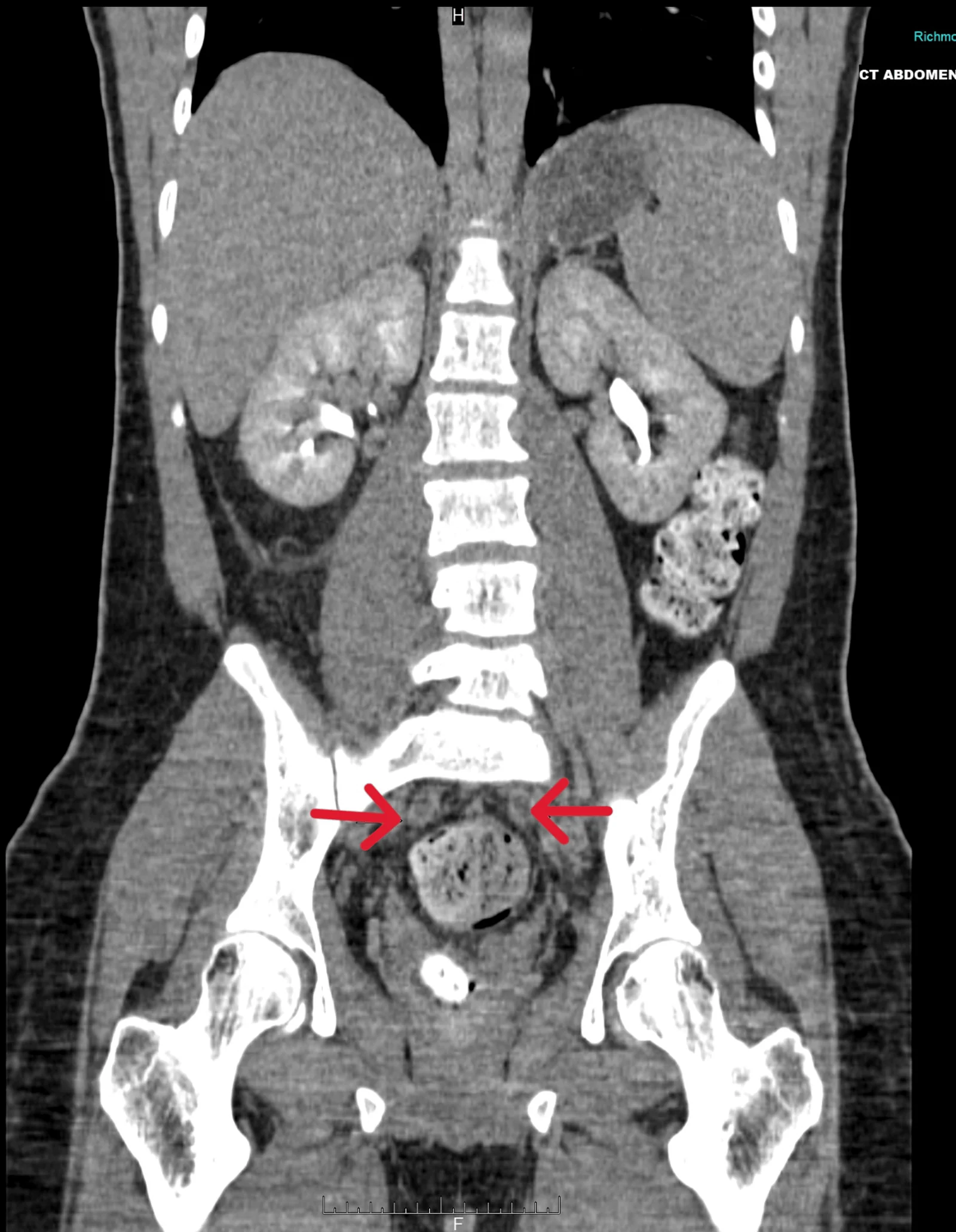
Initial coronal computed tomography angiography demonstrating extensive iliocaval thrombosis involving the pelvic venous system (red arrows)

**Figure 2:**
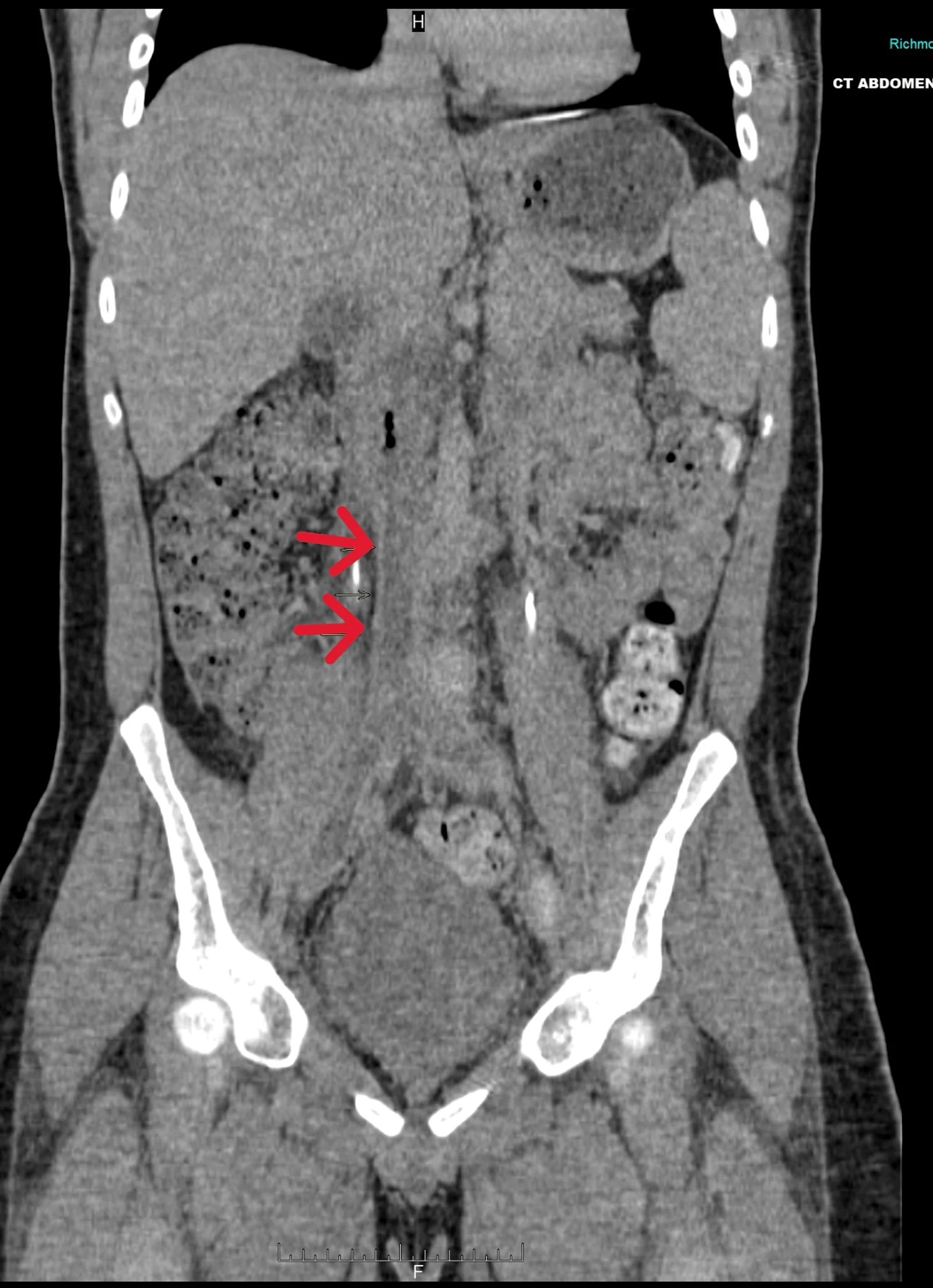
Coronal computed tomography angiography showing thrombus extending within the inferior vena cava (red arrows)

**Figure 3:**
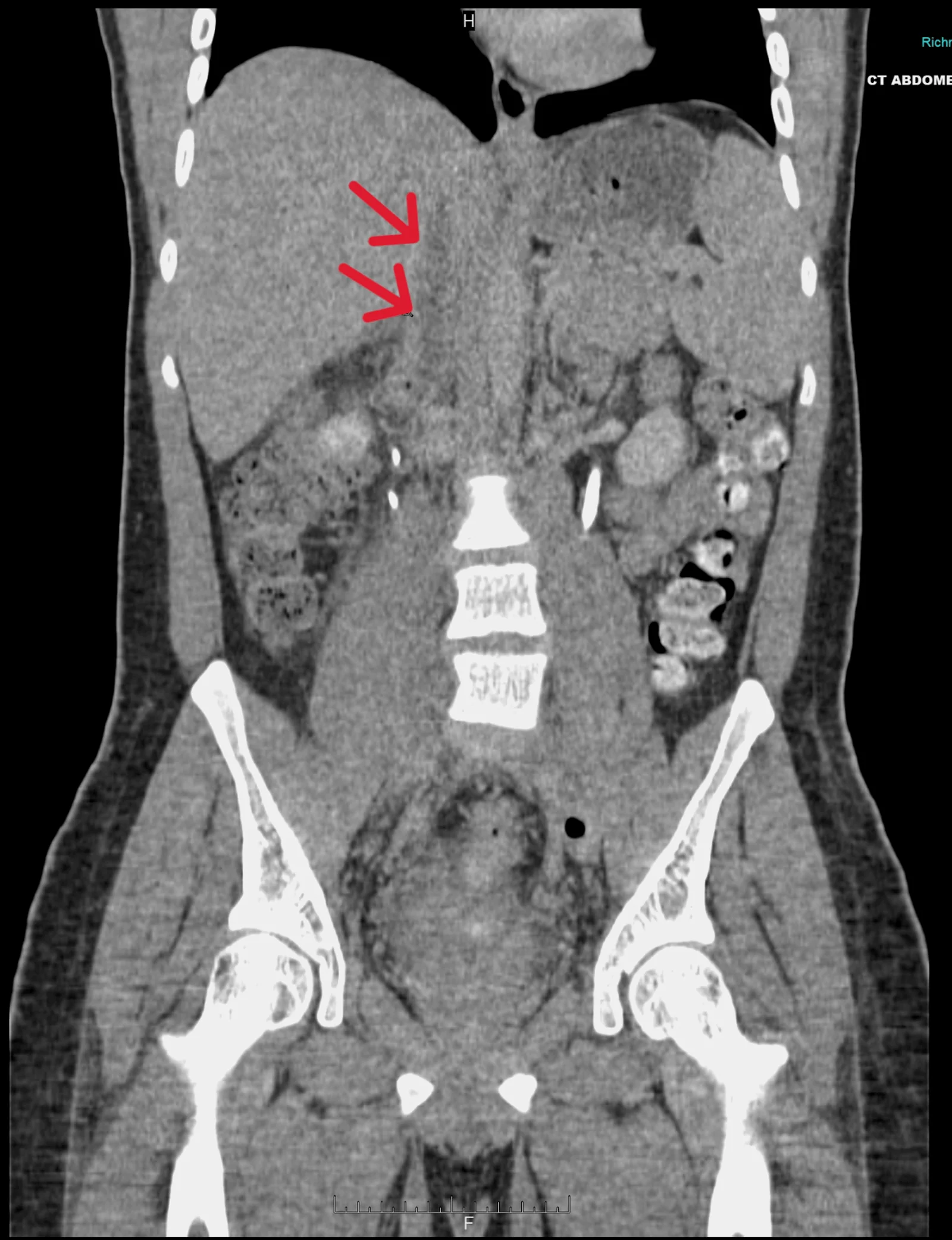
Coronal computed tomography angiography demonstrating extension into the intrahepatic inferior vena cava and right renal vein (red arrows)

**Figure 4:**
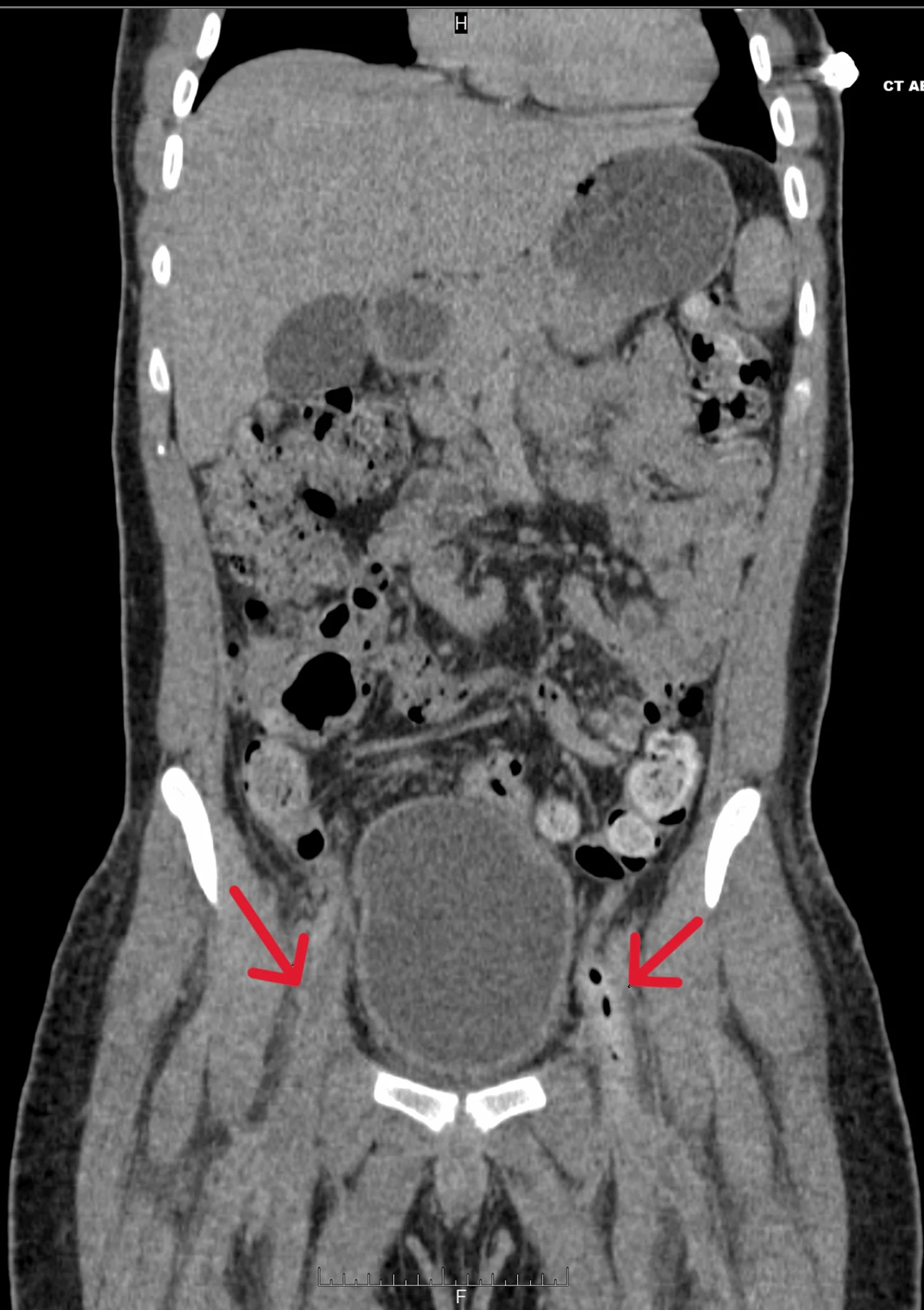
Coronal computed tomography angiography showing bilateral iliac/common femoral venous involvement (red arrows)

CTA of the abdomen and pelvis demonstrated extensive thrombotic occlusion involving the infrarenal IVC, with cephalad extension into the intrahepatic IVC, bilateral common and external iliac veins, and the right renal vein (Figures 1-4). Numerous enlarged lumbar, azygos, and paravertebral collateral veins were identified, suggesting chronic venous obstruction with superimposed acute thrombosis. CTA of the chest demonstrated no evidence of pulmonary embolism.

Therapeutic anticoagulation with continuous intravenous unfractionated heparin was initiated immediately after diagnosis. Given the extensive thrombotic burden, the involvement of the IVC and bilateral iliofemoral venous system, and the patient's young age, a multidisciplinary team involving vascular surgery, interventional radiology, and hematology recommended endovascular intervention.

Given the extensive iliocaval thrombus burden and acute abdominal and groin symptoms, endovascular intervention was pursued despite uncertainty regarding thrombus chronicity. The patient was maintained on a heparin infusion throughout the procedure. Right internal jugular venous access was obtained under ultrasound guidance, and a 24-French Inari Protrieve sheath (Irvine, California, United States) was advanced into the suprarenal IVC to provide proximal embolic protection. Cavography demonstrated a filling defect at the level of the renal veins corresponding to the thrombus identified on pre-procedural computed tomography. Aspiration/mechanical thrombectomy was subsequently performed. Only a small amount of whitish, chronic-appearing thrombus was retrieved despite multiple aspiration attempts. A specimen of the aspirated thrombus was submitted for culture given the patient's persistent fever.

Selective right iliac venography demonstrated extensive chronic-appearing thrombus extending from the common femoral vein to the iliac confluence, with prominent venous collaterals supporting a chronic component. Given the chronic appearance and limited thrombus extraction, further thrombectomy was discontinued, and anticoagulation was continued. No venoplasty was performed during this initial procedure. A suprarenal IVC filter was subsequently attempted because of the extensive thrombus burden; however, the filter failed to fully deploy, raising concern for an underlying venous web. After repositioning, the filter again failed to fully open and was subsequently removed. No further filter placement was attempted. Estimated blood loss was 50 mL, with no immediate procedural complications, and the patient remained clinically stable following the procedure.

Because of the persistent significant thrombus burden, a second endovascular procedure was subsequently performed with mechanical thrombectomy of the IVC and bilateral iliofemoral venous segments. Right internal jugular and bilateral femoral venous access was obtained, and an Inari ClotTriever system (Irvine, California, United States) was used for thrombectomy. Extensive chronic and subacute thrombus was removed from the IVC and bilateral iliofemoral segments. Repeat cavography demonstrated improvement in the caval and iliofemoral segments, although extensive residual thrombus remained. Stenoses identified within the IVC and bilateral iliofemoral segments were treated with balloon angioplasty using a 16-mm balloon in the IVC and 12-mm balloons in each iliofemoral segment, with resolution of the angiographic stenoses. Additional thrombectomy passes were performed bilaterally. Given the formation of a venous channel and the chronic nature of the residual thrombus, the procedure was discontinued. Overall, the intervention resulted in reduction of thrombus burden and channel formation but did not achieve complete thrombus clearance. The chronic-appearing thrombus, prominent venous collateralization, and procedural findings, together with the patient's acute abdominal and groin symptoms, were considered most consistent with an acute-on-chronic thrombotic process. However, the precise age of the thrombus could not be definitively established from imaging or procedural findings alone.

A comprehensive thrombophilia evaluation (Table 1) demonstrated homozygous FVL and reduced protein S activity (58%). Antithrombin III activity was normal (95%), testing for the prothrombin G20210A mutation was negative, and anticardiolipin and β2-glycoprotein I antibodies were negative. Lupus anticoagulant testing was positive; however, testing was performed during the acute thrombotic event while the patient was receiving anticoagulation, limiting its interpretation. Similarly, the reduced protein S activity was interpreted with caution because acute thrombosis, inflammation, and anticoagulation therapy may transiently decrease protein S levels. Therefore, the protein S result was not considered diagnostic of inherited protein S deficiency. Protein C activity and homocysteine levels were not obtained during the initial hospitalization and are reported as unavailable in Table 1. No other thrombophilic disorder was definitively established.

**Table 1:** Thrombophilia evaluation *Lupus anticoagulant testing was performed while the patient was receiving anticoagulation, which may affect test interpretation. IgG: immunoglobulin G; IgM: immunoglobulin M; IgA: immunoglobulin A

Investigation	Result	Interpretation
Factor V Leiden mutation	Homozygous positive	Abnormal
Protein S activity	58%	Reduced
Antithrombin III activity	95%	Normal
Prothrombin (factor II) G20210A mutation	Not detected	Negative
Anticardiolipin IgG antibody	<2.0	Negative
Anticardiolipin IgM antibody	<2.0	Negative
Anticardiolipin IgA antibody	<2.0	Negative
Beta-2 glycoprotein I IgG antibody	<2.0	Negative
Beta-2 glycoprotein I IgM antibody	<2.0	Negative
Beta-2 glycoprotein I IgA antibody	<2.0	Negative
Lupus anticoagulant	Positive*	Interpretation limited due to concurrent anticoagulation

Genetic testing performed during the admission confirmed homozygosity for the FVL variant, consistent with inheritance of one FVL allele from each parent. The molecular finding was consistent with the FVL R506Q variant. Given the confirmed homozygous FVL mutation and the patient's family history, homozygous FVL was considered the principal confirmed thrombophilic risk factor in this case. In the absence of malignancy, recent surgery, or trauma, prolonged international air travel with associated immobility was considered an additional transient provoking factor that may have contributed to the development of extensive venous thrombosis. The presence of extensive venous collateralization and chronic-appearing thrombus suggested that an underlying chronic venous outflow abnormality or longstanding venous obstruction may also have contributed; however, the precise underlying mechanism could not be definitively established.

Following intervention, the patient remained hemodynamically stable, with gradual improvement in lower extremity pain and swelling. He was continued on systemic anticoagulation during hospitalization and was discharged on therapeutic enoxaparin (Lovenox) 100 mg subcutaneously every 12 hours. Given the extensive proximal iliocaval thrombosis, young age at presentation, substantial residual thrombotic burden, and confirmed homozygous FVL thrombophilia, hematology recommended long-term, indefinite anticoagulation.

The patient was discharged with close follow-up with hematology and vascular surgery. At the three-month follow-up, he remained clinically stable with continued improvement in lower extremity symptoms and no reported recurrent VTE. No definitive imaging evidence of complete venous recanalization was available at that time. Given the chronic component and residual thrombosis identified during intervention, continued clinical surveillance and interval venous imaging may be considered based on symptoms and clinical course.

## Discussion

IVC thrombosis is an uncommon manifestation of VTE, accounting for fewer than 5% of lower extremity DVT cases [2,3]. Despite its rarity, iliocaval thrombosis carries significant morbidity due to extensive clot burden, increased risk of pulmonary embolism, post-thrombotic syndrome, recurrent thrombosis, chronic venous insufficiency, and long-term impairment in quality of life [1,2]. Compared with isolated femoropopliteal DVT, iliocaval thrombosis is more frequently observed in younger patients and is often associated with inherited thrombophilia, congenital venous abnormalities, malignancy, or major provoking factors [2,4,6].

Our patient represents an unusual case of extensive iliocaval thrombosis involving the infrarenal and intrahepatic IVC, bilateral iliac veins, and right renal vein in the setting of homozygous FVL mutation following prolonged international air travel. Several features distinguish this case from previously reported presentations. First, the thrombus extended beyond the typical iliocaval distribution to involve the renal vein, reflecting an exceptionally extensive thrombotic burden. Second, the patient presented with fever and syncope rather than classic unilateral lower extremity swelling, contributing to diagnostic uncertainty. Finally, the presence of extensive venous collaterals and organized thrombus with limited retrieval during intervention suggested an acute-on-chronic thrombotic process.

Given the patient's young age and extensive thrombotic burden, alternative congenital and acquired causes of iliocaval thrombosis were also considered. Congenital IVC anomalies, including agenesis or severe hypoplasia, are recognized risk factors for extensive DVT in young adults but were not identified on cross-sectional imaging in this case. May-Thurner syndrome was also considered; however, the patient's thrombosis was bilateral with extensive involvement of the IVC and right renal vein, making isolated left iliac vein compression less likely. Clinical features suggestive of Behçet disease, including recurrent oral or genital ulceration and ocular involvement, were absent. Likewise, there were no clinical or laboratory findings suggestive of a myeloproliferative neoplasm or paroxysmal nocturnal hemoglobinuria. In the setting of confirmed homozygous FVL mutation, strong familial predisposition, and recent prolonged air travel, these findings support inherited thrombophilia with a transient provoking factor as the most likely explanation for the patient's presentation.

FVL is the most common inherited thrombophilia among individuals of European ancestry and results from a G1691A single nucleotide substitution in the factor V gene, leading to resistance to activated protein C-mediated anticoagulant activity [5]. Under normal physiologic conditions, activated protein C limits thrombin generation through the proteolytic inactivation of factor V. The FVL mutation disrupts this regulatory pathway, resulting in persistent thrombin generation and a hypercoagulable state [5,7]. While heterozygous carriers have a three- to eightfold increased lifetime risk of VTE, homozygous individuals have a substantially higher risk, with studies demonstrating approximately 20- to 80-fold increased risk compared with the general population [5]. Homozygous carriers also tend to develop thrombosis at a younger age and may have increased rates of recurrent events and unusual thrombotic presentations [5].

In this case, prolonged immobilization during a 12-hour international flight likely served as the primary provoking factor. Long-distance air travel contributes to VTE risk through venous stasis, reduced calf muscle activity, dehydration, and hemoconcentration [7]. Although travel-associated thrombosis remains uncommon among healthy individuals, inherited thrombophilia significantly increases susceptibility, particularly following flights lasting more than four to six hours [7,8]. The combination of homozygous FVL mutation and prolonged immobilization likely created a synergistic pro-thrombotic state, resulting in extensive thrombosis in an otherwise healthy young adult.

An additional noteworthy aspect of this case was the atypical clinical presentation. Fever is an uncommon but recognized manifestation of acute thrombosis and may result from inflammatory cytokine release, including interleukin-6 and tumor necrosis factor-α, following endothelial activation and thrombus formation [11]. Because fever often directs clinicians toward infectious etiologies, VTE may not initially be considered, particularly in young patients without typical symptoms. Similarly, syncope is an unusual presentation of DVT and more commonly raises concern for cardiac causes or pulmonary embolism. Although pulmonary embolism was not identified in this patient, transient embolic events or hemodynamic changes before presentation may have contributed to the syncopal episode. Awareness of these atypical manifestations is essential to prevent delays in diagnosis.

Cross-sectional imaging was critical in defining the extent and chronicity of disease. CTA demonstrated thrombus extending from the infrarenal IVC to the intrahepatic segment with involvement of bilateral iliac veins and the right renal vein. The presence of extensive lumbar and azygos collateral circulation suggested pre-existing venous obstruction. This finding was supported by intra-procedural venography demonstrating organized thrombus and limited aspiration despite multiple mechanical thrombectomy attempts. Differentiating acute from chronic thrombus is clinically important, as chronic organized thrombus is less responsive to endovascular therapies due to fibrosis and endothelial remodeling [2,3].

Management of extensive iliocaval thrombosis remains challenging and often requires a multidisciplinary approach involving hematology, vascular surgery, and interventional radiology. Therapeutic anticoagulation remains the foundation of treatment and should be initiated promptly after diagnosis [9]. Current guidelines generally recommend anticoagulation alone for most proximal DVTs, while catheter-directed thrombolysis or mechanical thrombectomy may be considered in carefully selected patients with extensive iliofemoral thrombosis, severe symptoms, low bleeding risk, or limb-threatening venous obstruction [9,10].

In our patient, staged mechanical thrombectomy was pursued because of his young age, extensive clot burden, severe symptoms, and the goal of preserving long-term venous function. Although partial restoration of venous flow was achieved after thrombectomy and balloon venoplasty, substantial organized thrombus remained. This highlights an important clinical consideration: limited thrombus extraction during intervention does not necessarily indicate procedural failure but may reflect chronic thrombus organization. Recognizing this distinction is important because repeated aggressive interventions may increase procedural risk without providing additional clinical benefit [10].

Long-term anticoagulation is an important consideration in patients with homozygous FVL and extensive proximal thrombosis. Given the persistent thrombophilic state and high risk of recurrence, extended or indefinite anticoagulation may be appropriate when the bleeding risk is acceptable [5,10]. Direct oral anticoagulants have become increasingly utilized because of comparable efficacy to vitamin K antagonists, reduced monitoring requirements, and favorable safety profiles [10]. However, treatment duration and agent selection should remain individualized based on thrombotic risk, bleeding risk, patient preferences, and clinical circumstances.

To our knowledge, reports describing simultaneous thrombosis involving the intrahepatic IVC, bilateral iliac veins, and renal vein in a patient with homozygous FVL mutation remain limited. Additionally, the combination of fever and syncope as presenting manifestations of extensive iliocaval thrombosis is unusual. This case emphasizes the importance of maintaining a high index of suspicion for VTE in patients with atypical systemic symptoms, particularly when significant thrombotic risk factors are present. The coexistence of severe clot burden, inherited thrombophilia, prolonged air travel exposure, and evidence of acute-on-chronic thrombosis makes this case a unique contribution to the literature.

## Conclusions

This case highlights the importance of considering inherited thrombophilia in young patients presenting with extensive or atypical venous thrombosis, particularly when the extent of thrombosis appears disproportionate to common provoking factors. Homozygous FVL was identified in this patient with extensive iliocaval thrombosis following prolonged air travel. While the findings support an association between an underlying inherited thrombophilia and the patient's thrombotic presentation, causal or mechanistic conclusions cannot be established from a single case. Long-term anticoagulation should be individualized according to the patient's thrombotic and bleeding risks, clinical circumstances, and current guideline recommendations.
